# Nidoviruses in Reptiles: A Review

**DOI:** 10.3389/fvets.2021.733404

**Published:** 2021-09-21

**Authors:** Kate Parrish, Peter D. Kirkland, Lee F. Skerratt, Ellen Ariel

**Affiliations:** ^1^Virology Laboratory, Elizabeth Macarthur Agricultural Institute, New South Wales (NSW) Department of Primary Industries, Menangle, NSW, Australia; ^2^College of Public Health, Medical and Veterinary Sciences, James Cook University, Townsville, QLD, Australia; ^3^Faculty of Veterinary and Agricultural Sciences, Melbourne Veterinary School, University of Melbourne, Melbourne, VIC, Australia

**Keywords:** reptile, nidovirus, taxonomy, serpentovirus, respiratory disease, infectious disease

## Abstract

Since their discovery in 2014, reptile nidoviruses (also known as serpentoviruses) have emerged as significant pathogens worldwide. They are known for causing severe and often fatal respiratory disease in various captive snake species, especially pythons. Related viruses have been detected in other reptiles with and without respiratory disease, including captive and wild populations of lizards, and wild populations of freshwater turtles. There are many opportunities to better understand the viral diversity, species susceptibility, and clinical presentation in different species in this relatively new field of research. In captive snake collections, reptile nidoviruses can spread quickly and be associated with high morbidity and mortality, yet the potential disease risk to wild reptile populations remains largely unknown, despite reptile species declining on a global scale. Experimental studies or investigations of disease outbreaks in wild reptile populations are scarce, leaving the available literature limited mostly to exploring findings of naturally infected animals in captivity. Further studies into the pathogenesis of different reptile nidoviruses in a variety of reptile species is required to explore the complexity of disease and routes of transmission. This review focuses on the biology of these viruses, hosts and geographic distribution, clinical signs and pathology, laboratory diagnosis and management of reptile nidovirus infections to better understand nidovirus infections in reptiles.

## Introduction

The order *Nidovirales* is a large group of diverse enveloped positive-strand RNA viruses (1). Nidoviruses are known to infect a range of vertebrate and invertebrate hosts, several of which have caused serious diseases in both humans and animals. In humans, prominent nidoviruses belong to the family *Coronaviridae* and infections can result in a wide range of presentations from asymptomatic infections to significant morbidity and mortality associated with severe acute respiratory syndrome coronavirus (SARS-CoV) and Middle East respiratory syndrome (MERS-CoV) (2, 3). This family also includes the virus responsible for the current COVID-19 global pandemic, severe acute respiratory syndrome coronavirus 2 (SARS CoV-2) (4).

Following the emergence of these viruses in humans from animal sources, there is a renewed interest in animal nidoviruses including understanding the risk of cross species transmission from wildlife reservoirs. Novel viruses originating in wildlife reservoirs, especially bats, have also caused significant mortality and morbidity in animal populations, including swine acute diarrhoea syndrome coronavirus (SADS-CoV). This virus was implicated in the death of nearly 25,000 piglets (5). Other nidoviruses in animals associated with significant economic losses include infections with equine arteritis virus (EAV), porcine reproductive and respiratory syndrome virus (PRRSV), porcine epidemic diarrhoea virus (PEDV) and infectious bursal disease virus (IBDV) (6–8). Although most well-known nidoviruses are associated with terrestrial hosts, they also infect and cause significant disease in fish (white bream virus, fathead minnow virus, chinook salmon bafinivirus), shrimp (yellow head virus, gill-associated virus), and several lesser known nidoviruses infect sea hares, freshwater free-living flatworms, crabs, and marine mammals (9–12). Due to their increasing importance and recent association with morbidity and mortality, nidoviruses are also of interest in reptiles.

The number of viruses in the order *Nidovirales* continues to expand rapidly with the introduction of next generation sequencing (NGS) and metagenomics studies (13, 14). Such technology allows for an unbiased approach to pathogen detection when classical methods of diagnosis are unsuccessful. This is how nidoviruses in reptiles were first discovered and subsequently reported in 2014 (15–17). Respiratory disease in captive ball pythons (*Python regius*) is not a new phenomenon, with some groups reporting a syndrome of unknown cause being observed by veterinarians since the late 1990's (16). Following the exclusion of known pathogens, several research groups simultaneously used NGS to identify novel nidovirus sequences in captive pythons with respiratory disease (15–17).

Since 2014, additional reptile nidoviruses in snakes have been discovered globally in a wide range of predominantly Pythonidae species (13, 14, 18–20). They have also been found in lizards and turtles (21–23). Like the captive ball pythons, a respiratory syndrome has been reported in wild Australian shingleback lizards (*Tiliqua rugosa*) since the 1990's. In 2016, the first nidovirus in a lizard was reported in this species, both with and without respiratory disease (21). More recently another novel lizard associated nidovirus was discovered following respiratory disease-associated mortalities in a captive collection of veiled chameleons (*Chamaeleo calyptratus*) (23). In freshwater turtles, the only reported nidovirus was discovered following a mortality event in the sole extant wild population of the freshwater Bellinger River snapping turtle (*Myuchelys georgesi*). In contrast to the infections in snakes and lizards, respiratory disease was not the predominant syndrome observed, but rather the most significant pathological changes were in the kidneys (22).

As the number of nidovirus detections in reptiles continues to grow, for the most part, studies are confined to captive collections, especially of pythons. Consequently, the disease risk that reptile nidoviruses pose to wild populations of reptiles is largely unknown. Worldwide the number of reptile species listed as threatened continues to increase (24) and emerging or reemerging infectious diseases including various fungi (25–27), bacteria (28) and viruses (29–31) are cause for concern. Infectious disease is rarely a single contributing factor in known plant and animal extinctions, with the exception being amphibian panzootics caused by chytrid fungus (*Batrachochytrium dendrobatidis*) (32–34), yet understanding the risk they pose may be critical in preventing ongoing population declines. This relatively new field of research offers unique opportunities to explore major gaps in knowledge. This review summarises the key findings to date from the published literature and offers recommendations for the direction of future research.

## Materials and Methods

**Figure 1:**
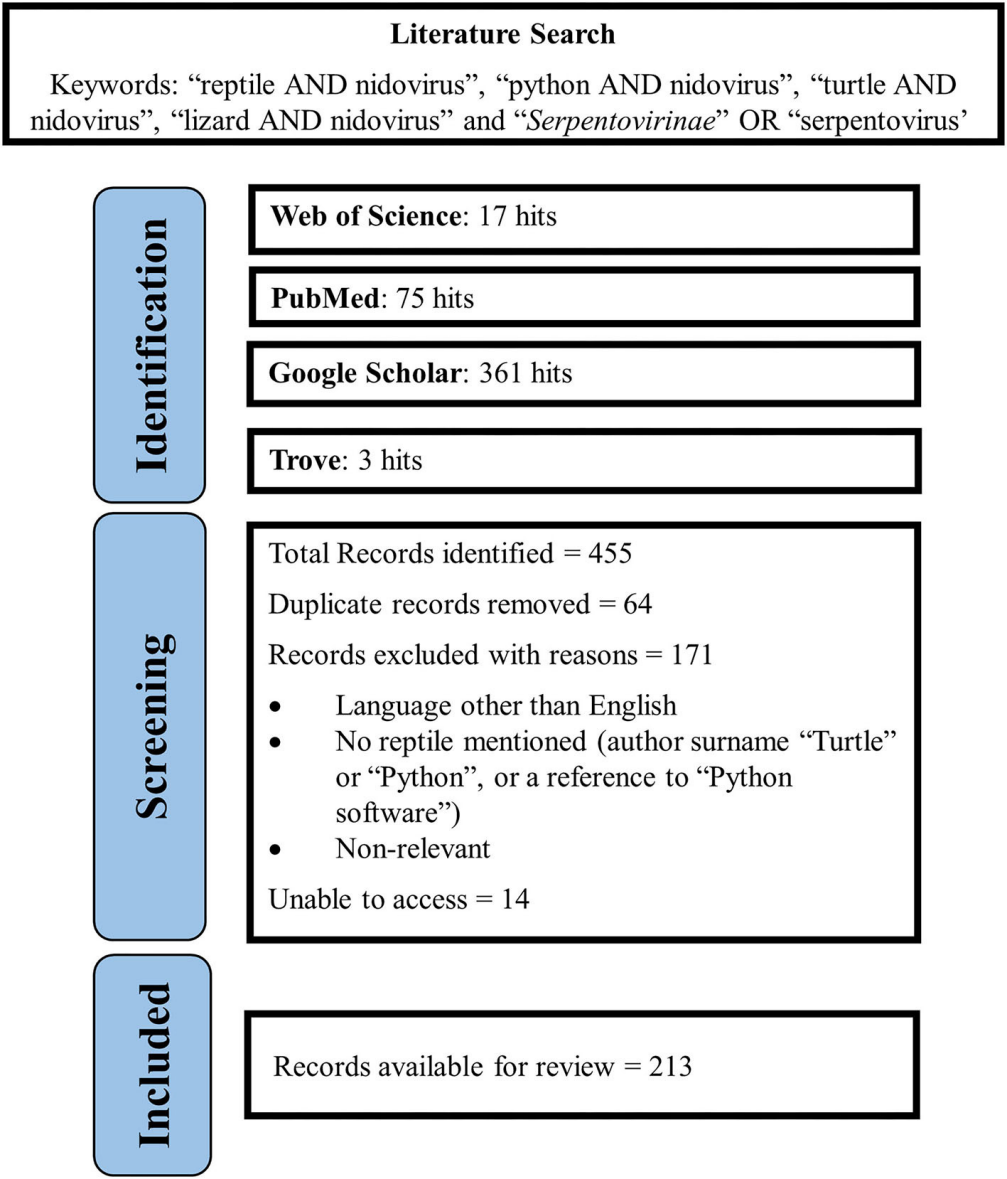
Literature search methodology.

A literature search was conducted between January 2018 and June 2021 using the following databases: Web of Science (Clarivate Analytics), PubMed, Google Scholar to find peer-reviewed articles as well as Trove for thesis manuscripts. Search terms included “reptile AND nidovirus,” “python AND nidovirus,” “turtle AND nidovirus,” “lizard AND nidovirus,” and “*Serpentovirinae*” OR “serpentovirus.” Searches were conducted without limits on publication dates or geographical location. A total of 455 articles were identified and 391 remained once duplicates were removed. Additional articles were excluded if the study was not available in English, a reptile was not referred to throughout the article (only an author surname or Python software© or programming language), unable to access or non-relevant articles leaving 213 articles to be reviewed. Additional articles were found manually from the citations of relevant articles. A summary of the search terms and results can be found in Figure 1.

**Figure 2:**
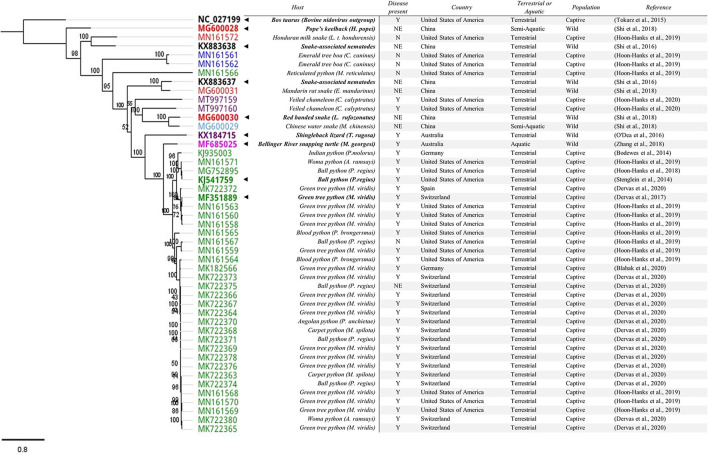
Phylogenetic tree of reptile nidovirus sequences with >10,000bp. The entirety of ORF 1b amino acid sequences were aligned. Following initial alignment three sequences were removed (MK182569, MK722379, and MK722377) where they had 100% similarity to corresponding sequences that were included (MK18256, MK722366, and MK722376), leaving 47 sequences (including bovine nidovirus (NC_027199) in the alignment. Genbank accession numbers are included in the tree alongside key features in the table. The current ICTV approved members of subfamily ***Serpentovirinae*** are **BOLD** with a “◂” to highlight them. The associated host is also in **BOLD**. The presence of disease is reported as Y = Yes, N = No, NE = Not Examined. For snakes, host families are indicated in the tree: Pythons (Pythonidae, green), Boas (Boidae, blue), Colubrids (Colubridae, red), and Homalopsid (Homalopsidae, light blue). The remaining sequences from reptiles including lizards (Chamaeleonidae and Scincidae, purple) and turtles (Chelidae, pink) are also coloured. The nematode associated sequences and the bovine nidovirus sequence remain uncoloured.

To identify key published *Serpentovirinae* spp. sequences, viruses identified by the International Committee on Taxonomy of Viruses (ICTV) (*n* = 8) were included (Table 1) as well as sequences where a substantial proportion of genome has been sequenced. Sequences were included if they had more than 10,000 base pairs (bp) and were sequenced from a reptile or reptile-associated sample (*n* = 41). The following search terms were used to identify these sequences; “*Tobaniviridae*,” “*Serpentovirinae*,” “unclassified *Nidovirales*,” “unclassified *Torovirinae*,” and “unclassified *Serpentovirinae*” (Supplementary Table 1). A phylogenetic tree of reptile nidovirus sequences (*n* = 49) and a remotovirus (bovine nidovirus) from the family *Tobaniviridae* can be found in Figure 2. The entirety of ORF 1b amino acids were aligned using Geneious Prime® (Version 2021.1.1) and based on these alignments maximum likelihood trees (PhyML) were calculated using the HKY85 substitution model and 1,000 bootstrap replicates. Following initial alignment, three sequences were removed (MK182569, MK722379, and MK722377) where they had 100% similarity to corresponding sequences that were included (MK182566, MK722366, and MK722376) leaving 47 sequences including bovine nidovirus (NC_027199) in the alignment (Figure 2).

**Table 1:** Viruses within the subfamily *Serpentovirinae* (35).

**Genus**	**Subgenus**	**Species**	**Virus name**	**Accession**	**Genome coverage**	**Host**	**Country**	**Size (nt)**	**References**
Pregotovirus	*Roypretovirus*	*Ball python nidovirus 1*	Ball python nidovirus (BPNV)	KJ541759	Complete genome	Ball python (*P.regius*)	USA, CHE	33, 452	(16)
		*Morelia tobanivirus 1*	Morelia viridis nidovirus (MVNV)	MF351889	Complete genome	Green tree python (*M. viridis*)		32, 399	(18)
	*Snaturtovirus*	*Berisnavirus 1*	Bellinger River virus (BRV)	MF685025	Complete genome	Bellinger River snapping turtle (*M. georgesi*)	AUS	30, 742	(22)
	*Tilitovirus*	*Shingleback nidovirus 1*	Shingleback nidovirus (SBNV)	KX184715	Partial genome	Shingleback lizard (*T. rugosa*)	AUS	23, 832	(21)
Sectovirus	*Sanematovirus*	*Sectovirus 1*	Xinzhou nematode virus 6	KX883637	Partial genome	Snake-associated nematodes mix Xinzhou [Nematoda spp. (14), Ascarididae spp. (2)]	CHN	25, 960	(13)
Infratovirus	*Hepoptovirus*	*Hebius tobanivirus 1*	Hainan hebius popei torovirus	MG600028	Complete coding genome	Pope's keelback (*H. popei*)	CHN	29, 409	(14)
	*Xintolivirus*	*Infratovirus 1*	Xinzhou toro-like virus	KX883638	Complete coding genome	Snake-associated nematodes mix Xinzhou [Nematoda spp. (14), *Ascarididae* spp. (2)]	CHN	30, 353	(13)
Lyctovirus	*Rebatovirus*	*Lycodon tobanivirus 1*	Guangdong red-banded snake-Lycodon rufozonatus-torovirus	MG600030	Complete coding genome	Red banded snake (*L. rufozonatus*)	CHN	30, 859	(14)

*Country of origin: United States of America (USA), Switzerland (CHE), Australia (AUS), China (CHN)*.

## Taxonomy

Prior to 2019, the order *Nidovirales* was composed of four families: *Arteriviridae, Coronaviridae, Mesoniviridae*, and *Roniviridae* (36). At the time of their discovery, the group of reptilian nidoviruses clustered within a proposed new genus within the family *Coronaviridae* and subfamily *Torovirinae* (16, 18, 22). Recently, a new taxonomic nomenclature was approved by the ICTV. This revised taxonomy has resulted in 8 suborders, 14 families, 25 subfamilies, 39 genera, 65 subgenera and 109 species (35, 37). This reclassification has now placed reptile-associated nidoviruses within the suborder *Tornidovirineae* and the family *Tobaniviridae*. The nidoviruses discovered in snakes, turtles and lizards have been grouped into the subfamily *Serpentovirinae* (Table 1) (35). This has led to reptile-associated nidoviruses, potentially misleadingly, being referred to as “serpentoviruses” (20). Derived from the Latin word “serpēns,” the term “serpent” is synonymous with “snake” for many people and it is important not to categorise these viruses as only “snake” viruses. With an expected increase in the discovery of novel reptile nidoviruses, there will likely be additional changes to classification in the future.

## Biology of the Virus

### Virus Morphology and Size

*Nidovirales* exhibit significant diversity in their morphology. The recently revised family *Tobaniviridae* includes both of the previously known genera *Torovirus* and *Bafinivirus*. When examined by electron microscopy, toroviruses are known to be pleomorphic with bacilliform (rod-shaped), kidney shaped, and spherical virions often observed, while bafiniviruses have a rod-like or bacilliform appearance. A distinctive feature of both the previously known toroviruses and bafiniviruses are the surface projections that correspond to the club or petal shaped projections found on the surface of coronaviruses (36, 38).

Within the *Serpentovirinae* subfamily, Stenglein et al. (16) identified ball python nidovirus (BPNV) particles within the pneumocytes of affected lung tissue. They were able to capture the pleomorphic appearance of the virus representing the various stages of viral replication. Mature bacillary virions measured 180 × 50 nm while the uncoated intracellular viral capsids measured 10–12 nm. The bacillary nucleocapsids contained a lucent core that was surrounded by fine granular cytoplasmic material presumed to be a component of the envelope. A cross section of a mature virion demonstrated a clear lipid envelope and surface spikes within cytoplasmic vesicles (16). In cell culture supernatant, Bellinger River virus (BRV) virions were bacilliform in appearance (22). They measured 170 nm long while Morelia viridis nidovirus (MVNV) had both a rod and kidney shaped appearance, measuring ~120 nm in length (100–150 nm) (18, 22). Both BPNV and MVNV bacillary particles have been observed as abundant tubular structures arranged in a stack formation within the cytoplasm of affected cells (16, 18). In contrast to the morphological descriptions available for the broader order *Nidovirales*, descriptions within the *Serpentovirinae* subfamily are similar, but limited to a few publications.

### Genome (Size), Structure, and Protein Expression

Historically nidoviruses were grouped into small and large, with the small nidoviruses originating from a single family, *Arteriviridae*, with genomes of 12.7–15.7 kb in length. With the revised taxonomy, there are now three other families that have genomes <15 kb (35). Viruses belonging to the family *Tobaniviridae*, in the order *Nidovirales*, continue to be some of the largest RNA genomes known (1, 11). At the time of discovery, BPNV was the largest known RNA virus with a 33.5 kb viral genome, however in 2018, planarian secretory cell nidovirus (PSCNV), about 25% larger with a 41.1 kb genome, was discovered in a free-living flatworm (11). Despite the wide variation in genome size, the nidoviruses generally share a similar genome structure. They are typically made up of multiple open reading frames (ORFs), including overlapping ORF 1a and ORF 1b, and multiple ORFs at the 3′-end, which are flanked by both the 5′-end untranslated region (UTR) and the 3′-end UTR (1).

Nidoviruses in reptiles are known to share this genome structure, with two large overlapping 5′-end ORFs, replicase ORF 1a and 1b, with a ribosomal frameshift signal (-1, AAAAAC) (15–18, 21, 22). These encode two polyproteins: pp1a and pp1ab that are known to be involved in viral genome replication, expression and modulation of host cell activities (1, 39). The production of the large pp1ab is a key feature of nidoviruses, as is a set of functional subunits within this protein. Depending on the study and the amount of genome sequenced (partial or full), the pp1ab and most of the associated functional subunits have been identified in reptile nidoviruses (15, 16, 18, 21–23).

Reptile nidoviruses share the distinct replication strategy for viruses within the order *Nidovirales*. Named from the Latin word “nidus” for nest, the order is characterised by a nested set of viral subgenomic messenger RNA's that are produced during infection (1, 3, 40). In reptile nidoviruses, are several ORF's at the 3′-end coding for the “S” or spike glycoprotein (ORF 2), and other structural proteins including the transmembrane glycoproteins, matrix protein and nucleocapsid protein. These proteins are expressed from the subgenomic messenger RNA's (15–17, 22). With ongoing research into reptile nidoviruses we anticipate more studies will be published on the impact of variations of these proteins on viral pathogenesis or virulence.

### Viral Diversity

Divergent nidoviruses have been discovered in different snake species. The initial reptile nidovirus sequences found in the ball python, *P. regius* (16, 17) and the Indian python, *P. molorus* (15) were relatively similar. Subsequent partial and full-length sequences that were detected in the python genus *Morelia spp*. were genetically different (18, 19). In 2017, a complete genome of MVNV was sequenced from a green tree python (*M. viridis*) in Switzerland. This sequence was <85% identical to BPNV (18). Additional sequences from *M. viridis* have continued to show diversity, with two complete genomes from Germany that share a 99.7% nucleotide identity with each other, but only 66.8 and 66.9% overall similarity to the original MVNV isolate from Switzerland (41).

Generally, virus sequences from Pythonidae (pythons) tend to cluster (Figure 2). To date, a clear host species-specific lineage is not evident. In one collection, virtually identical sequences (≥99.8% pairwise identity) were identified in three different snake genera within the family Pythonidae: *Morelia* spp., *Antaresia* spp., and *Python* spp. (20) suggesting similar viruses are capable of infecting multiple python species. Sequences found in Boidae (boas), Colubridae (colubrids), and Homalopsidae (mud snakes) are genetically different when compared to those found in Pythonidae (14, 19, 20). The exception are sequences found in reticulated pythons (*M. reticulatus*) that clustered with sequences found in boas, colubrids and mud snakes (Figure 2) (20).

In contrast, our knowledge of the reptile nidovirus diversity in lizards and turtles is limited to key publications with viruses detected from several reptile families including Scincidae (skinks), Chamaeleonidae (chameleons), and Chelidae (Austro-South American side-neck turtles) (21–23). Despite the apparent clustering of similar viruses in certain snake families, further research is required to determine if there are clear host specific nidovirus lineages, especially in the lesser studied species and wild reptile populations. It is likely our discovery of reptile nidovirus diversity is just beginning.

## Hosts and Geographic Distribution

Reptile nidoviruses have been detected worldwide. This includes detections on four continents, including North America, Europe, Asia, and Australia. They have also been found in multiple hosts from the orders Squamata (lizards and snakes) and Testudines (turtle, tortoise and terrapin).

### Squamata (Lizards and Snakes)

The frequency of nidoviral detections in snake species has increased dramatically in recent years. This is largely a result of polymerase chain reaction (PCR) based surveys of captive snake species (19, 20, 41–43). To date, viruses have been discovered in several snake families including Pythonidae, Boidae, Colubridae, and Homalopsidae (14, 19, 20, 41). Nidoviral prevalence in captive python species has been reported to be as high as 27.4% (19), 30.7% (41) and 37.7% (20). Others have also reported differences in prevalence between species, with detections occurring more frequently in the green tree python (*M. viridis*) (32.2, 41.2, 75.8%) when compared with the ball python (*P. regius*) (22.2, 22.1, 5.1%) (20, 41, 42). Infections are more frequent in pythons, however, sampling has been weighted toward Pythonidae species.

In contrast to Pythonidae, the prevalence in snakes from the Boidae and Colubridae families appears to be significantly less with 2.4, 10.1, and 0.8% reported in captive Boidae species (19, 20, 41) and 0.9% reported in captive Colubridae species (20). However, much lower sample numbers have been used to generate these values. It is also possible that genetically diverse nidoviruses in Boidae and Colubriae families may have been missed with the current assay designs. Metagenomic sequencing of negative samples could minimise this possibility (44). Only limited sampling of wild snake species has been undertaken and only as part of a large scale meta-transcriptomics survey to detect vertebrate associated RNA viruses (14). This survey identified key reptile nidovirus sequences found in snakes from the families Colubridae and Homalopsidae in China but did not report on prevalence or presence of disease. In the future, surveys to obtain an unbiased estimate of prevalence in captive and wild snake populations are required.

O'Dea et al. (21) first reported shingleback nidovirus 1 (SBNV) in Australian shingleback lizards (*Tiliqua rugosa*) admitted to a wildlife rehabilitation centre. The virus was present in lizards with and without respiratory disease. Ongoing surveillance at two wildlife rehabilitation centres found 58.1% of *T. rugosa* admitted to this facility (for a variety of reasons) gave positive results in an SBNV PCR (45). Surveys of wild shingleback populations (not those submitted to a care facility) have not been undertaken to date. Two novel nidoviruses have recently been described in a collection of veiled chameleons (*Chamaeleo calyptratus*) experiencing respiratory disease associated mortalities (23). The two genotypically distinct viruses were named veiled chameleon serpentovirus A (VCSV-A) and B (VCSV-B). Additional lizard species were housed in the same facility, including bearded dragons (*Pogona vitticeps*), common leopard geckos (*Eublepharis macularius*), and ocelot geckos (*Paroedura pictus*). These animals were clinically healthy throughout this period and gave negative results for VCSV by PCR (23). This could be due to a lack of exposure or resistance to VCSV infection. Opportunistic PCR based surveys of wild or captive lizards could provide insight into the range of lizard species susceptible to infection with reptile nidoviruses. Following on from this, similarly to snakes, random surveys to obtain unbiased estimates of prevalence in captive and wild snake populations are required.

### Testudines (Turtles, Tortoises, and Terrapins)

The first report of a nidovirus, BRV, in a turtle was in 2015. It was detected in a wild population of freshwater turtles (*M. georgesi*), in a single river system, and was implicated in the mortality of over 400 turtles (22). This river system is home to many other species including reptiles, amphibians, arthropods, and fish. No morbidity or mortality was reported in any other species at the time, including the sympatric Murray River turtle (*Emydura macquarii*). Since 2015, there have been no confirmed clinical cases of BRV in wild *M. georgesi*, yet BRV was detected on conjunctival swabs from a small number of clinically normal *M. georgesi* (9 of 31 sampled) and *E. macquarii* (2 of 49 sampled) in a follow-up survey 6 months after the cessation of the outbreak (22). In addition, swabs (*n* = 360) from many reptiles, amphibians, arthropods, and fish failed to detect other species infected with BRV (22). Ongoing PCR based surveys to provide insight into the prevalence, incidence, and clinical outcomes of apparently asymptomatic individuals following this mortality event are required. Opportunistic sample collection associated with population monitoring surveys presents a cost-effective option for infectious disease detection and monitoring and could be considered for other reptile species (46, 47).

### Other Species

Closely related nidoviruses have been detected in snake-associated nematodes as part of a large scale metagenomic screening of vertebrate and invertebrate samples (13, 14). The significance of these detections remains uncertain. The nematodes may have ingested or been contaminated with a nidovirus infecting the snake. While unlikely, the possibility of a recent horizontal transfer between species cannot be excluded (48). Additional testing of nematodes is required to clarify their susceptibility to nidovirus infection.

## Clinical Signs, Pathology, and Tissue Tropism

### Clinical Signs

Nidoviruses are known for causing respiratory and enteric disease in terrestrial vertebrates (49). This is true for viruses infecting cattle, horses, chickens, and pigs (6, 50–52). In aquatic animals, while not as well-studied only some nidoviruses appear to follow this trend (12, 53, 54). To provide an overview, the clinical signs reported to be associated with reptile nidovirus infections are summarised in Table 2. Clinical signs appear in order from the most reported to the least reported across species from key reptile nidovirus publications.

**Table 2:** Clinical signs associated with nidoviruses in reptiles.

**Clinical sign**	**Squamata**									**Testudines**	**References**
Secretion from oral cavity of clear, foamy, mucoid or mucopurulent material	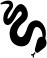	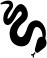	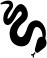	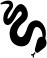	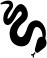	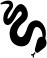	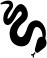	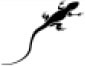	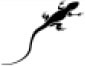		(16, 18, 20, 21, 23, 41, 55–57)
Found dead or sudden death	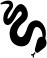	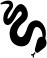	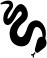	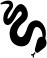	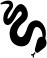	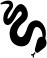		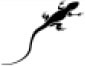		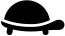	(15–18, 22, 23, 57, 58)
Dyspnea or open mouth breathing including increased respiratory rate (respiratory distress)	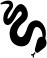	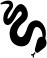	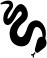	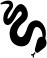	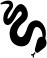	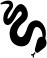		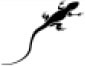			(16, 18, 20, 23, 41, 55, 57)
Hyperaemia or inflammation of mucous membranes in the oral cavity	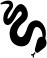	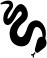	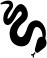	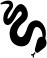	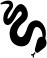	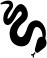					(16, 17, 20, 41, 55, 59)
Anorexia or inappetence	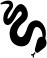	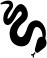	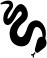	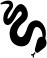	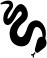			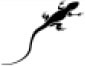			(16, 18, 20, 23, 55, 57)
Audible breathing or wheezing	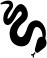	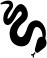	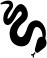					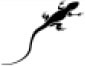			(16, 20, 23, 56)
Secretion of clear, foamy, mucoid or mucopurulent material from nasal passages	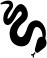	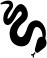						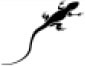		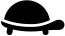	(20–22, 57, 60)
Poor body condition or weight loss	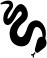							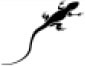	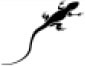	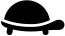	(21–23, 56)
Notable “cough-like” forced expiration	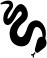	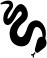									(16, 20)
Ocular discharge (clear to mucopurulent)								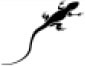	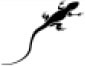		(21, 45)
Lethargy or depression								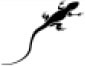		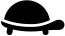	(21, 22)
Expulsion of mucus	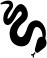										(18)
Excessive swallowing	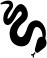										(55)
Ventral oral swelling	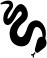										(55)
Spectaculitis	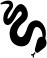										(20)
Petechiations (small oral mucosal haemorrhages)	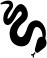										(55)
Opisthotonos (star gazing)	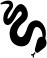										(16)
Emaciation	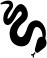										(18)
Inappropriate shedding	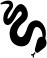										(20)
Difficulty perching in arboreal snakes	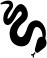										(20)
Bilateral crusting of the eyes								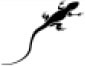			(23)
Sunken eyes								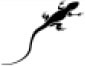			(23)
Pale mucous membranes								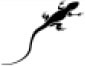			(21)
Sneezing								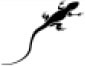			(21)
Vertical head tilt								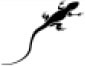			(23)
Reduced water intake								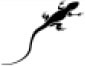			(23)
Severe bilateral ocular inflammation										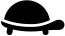	(22)
Hindlimb paresis										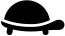	(60)
Tan foci on skin of ventral thighs										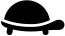	(22)

*The clinical signs associated with nidovirus infected reptiles reported from key reptile nidovirus publications are included. Included publications discovered or conducted research on reptile nidoviruses specifically. Host species are classified as Squamata; snakes (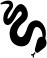), lizards (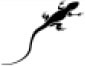), and Testudines; turtles (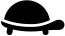). A single publication where “pneumonia” was described as a clinical sign, has been included under “Dyspnea or open mouth breathing including increased respiratory rate (respiratory distress)” which likely would have been observed clinically (41). Clinical signs appear in order from the most reported across species to the least reported*.

In reptiles, clinical signs associated with infection of the respiratory tract appear to be the most common feature of nidovirus infection (15, 16, 18, 21, 41, 55). Initial clinical signs in captive pythons include increased amounts of clear or mucoid material in the nose and mouth and oral inflammation (stomatitis). This proceeds to wheezing, open mouth breathing, increased respiratory rate, or coughing. Additional clinical signs include inappetence, weight loss, lethargy, dehydration, inappropriate skin shedding, difficulty perching in arboreal snakes, and speculitis (20, 56). In some cases, a respiratory syndrome characterised by severe acute pneumonia and sudden death has occurred (15–18, 58). In a PCR based nidovirus survey of captive snakes, clinical signs of respiratory disease were more common in infected pythons (85 of 144) compared to boas (1 of 8) (20). This may suggest differences in species susceptibility or differences in nidovirus virulence.

A single study has confirmed Koch's postulates using a reptile nidovirus and described the clinical signs observed following infection. In 2018, three captive bred ball python (*P. regius*) juveniles (~6 weeks old) were exposed orally and intratracheally with a cell culture grown BPNV. Clinical signs were observed 4 weeks after exposure and included mucosal hyperaemia and profuse mucus secretion, followed by a progression to the appearance of petechial mucosal haemorrhages, open mouth breathing, and anorexia by 10–12 weeks post exposure. The presence of infectious virus was confirmed using virus isolation from oroesophageal swabs taken on the day of euthanasia; 5, 10, and 12 weeks post exposure (55).

Australian shingleback lizards (*T. rugosa*) infected with SBNV can also present with respiratory disease. Similarly to *P. regius*, a respiratory syndrome has been observed since the 1990's that is characterised by excess mucous in the oral cavity, sneezing, serous to mucopurulent discharge from the eyes and nose, lethargy, inappetence, pale mucous membranes, depression, and emaciation (21). In captive bred chameleons (*C. calyptratus*) infected with VCSV, clinical signs included wheezing, vertical head tilting with gasping, increased mucus in the oral cavity, anorexia, and reduced water intake (23). To date, experimental infection studies to confirm the role of nidovirus in the development of respiratory disease has not been described in lizards.

Bellinger River snapping turtles (*M. georgesi*) infected with BRV were largely found as dead or moribund animals with bilateral ocular inflammation, poor body condition, and some had tan foci on the skin of the ventral thighs or hind limb paresis (22). Many also had a slight clear nasal discharge, and some animals had hindlimb paresis (60). In contrast to other species, respiratory disease was not the dominant syndrome observed. As with lizards, experimental infection trials have not yet been completed.

### Pathology and Tissue Tropism

Most pathology associated with nidoviral infection in snakes has been associated with the respiratory tract, and to a lesser extent the oral cavity and upper alimentary tract. The tropism for respiratory epithelium has also been confirmed using *in situ* hybridisation (ISH) (15, 18). Experimental infection of *P. regius* resulted in histological findings consistent with a chronic-active mucinous rhinitis, stomatitis, tracheitis, oesophagitis, and proliferative interstitial pneumonia (55). The proliferative interstitial pneumonia has been a consistent finding in clinical cases of nidovirus infection in snakes and has more recently been called “nidovirus associated proliferative disease—NPD” (16, 57).

Consistent with this pathology, the viral load is often highest in the lung tissue. However, the viral load in the intestine has also been reported at similar levels (16). This finding is consistent with other closely related viruses, namely coronaviruses and toroviruses, where respiratory and enteric tropism has been well-established (4, 61, 62). In green tree pythons (*M. viridis*) high viral loads have also been confirmed in the lung, but in contrast to the findings of Stenglein et al. (16) intestinal samples were mostly negative by PCR or negative using ISH (18, 41). This finding could be reflective of differences in tropism between reptile nidoviruses, susceptibility of different host species, or a function of the time of exposure and disease progression.

Since their discovery, nidoviruses have been reported in tissues other than the respiratory tract but their presence and pathology have been inconsistently examined and reported. Stenglein et al. (16) identified virus in liver, kidney, heart, spleen, and brain, but largely at levels 3–5 orders of magnitude lower than the lung. More recently, during multiple necropsies (*n* = 30) Dervas et al. (57) identified pyogranulomatous and fibrinonecrotic lesions in organ systems aside from the respiratory tract suggesting a much broader cell and tissue tropism. Virus was also detected in epithelial cells (alimentary, hepatic, renal, pancreatic), intravascular monocytes, intralesional macrophages, and endothelial cells (57). Dervas et al. (57) also identified animals with evidence of disseminated granulomatous and/or fibrinonecrotic lesions, vascular and perivascular lesions, and infected monocytes. These lesions were more predominant in *Morelia* spp. (57).

The suggestion of a broad cell and tissue tropism is consistent with the pathology caused by BRV in *M. georgesi*. There was histological evidence of fibrinonecrotising splenitis and nephritis with multisystemic fibrinoid vasculopathy (22, 60). Using ISH, BRV was detected in glandular epithelial cells, in areas of necrotising inflammation within the lacrimal gland, in degenerate or necrotic renal tubule epithelial cells, and in foci of vasculitis. Virus was also detected in necrotizing lesions in the urinary bladder, scattered granulocytes in the oedematous urothelium, and occasional granulocytes within the myocardial interstitium (22).

To date, no histology has been reported following infection of shingleback lizards (*T. rugosa*) with SBNV. However, a single veiled chameleon (*C. calyptratus*) coinfected with VCSV-A and VCSV-B had both respiratory symptoms and histological lung lesions like those identified in snakes. This included an interstitial proliferative and catarrhal pneumonia, rhinitis, and tracheitis (23). Interestingly, in this collection other VCSV infected veiled chameleons examined had no histologic lesions (*n* = 3) or mild non-specific lesions (*n* = 3) including focal xanthomatous mural enteritis with coelomic foreign body, severe heterophilic enteritis with mural granulomas, splenic lymphoid hyperplasia, mild lymphocytic portal hepatitis, rare mineralization of the tunica intima of large cardiac vessels, and hepatocellular vacuolization (23).

Transmission studies with reptile nidoviruses are scarce, with only a single experimental infection trial using BPNV and a small number of juvenile ball pythons (*P. regius*) (55). Subsequent studies have identified a statistically significant association between age and infection status, reporting that in a survey of captive snakes, older snakes were more likely to be infected, but that increasing age did not increase the likelihood of disease (20). This finding highlights the need for additional transmission trials to explore the “triad” of disease determinants: host, agent, and environment (63). For example, these trials could examine the impact of viruses from different backgrounds (cell culture amplified, passage level, strains), various host factors (age, species), routes of exposure, environmental temperatures, and the duration of the trial. This will allow for further assessment of the general pathology associated with nidovirus infections in reptiles. In the absence of this, we are left to make judgements of pathogenesis based on antemortem (clinical signs) and post-mortem findings of naturally infected animals.

### Asymptomatic Infection

The detection of a virus or nucleic acid in an animal without clinical signs could be a result of testing during the incubation period, the animal having recovered but continuing to be a carrier, or that some individuals remain asymptomatic following infection. The possibility of superficial contamination, ingestion, or inhalation of nucleic acid from the environment without active virus replication must also be considered. There is evidence that reptiles can be asymptomatic when infected with nidoviruses. Detections have been reported in snakes, lizards and turtles in the absence of clinical disease. In one survey of captive snakes, signs of respiratory disease were only identified in 59% (85 of 144) of infected pythons, 12.5% (1 of 8) of infected boas, and were absent in a single infected colubrid (20). Another survey that examined a large number of pythons that had given positive results in a PCR found only 17.8% (67 of 377) had stomatitis and/or respiratory disease (41) and a smaller survey in Poland found only 23.1% (3 of 13) of nidovirus positive pythons had respiratory disease at the time of testing (43). There is also some evidence that animals may remain infected and asymptomatic for prolonged periods. Five pythons (*Morelia spp*.) that were nidovirus PCR positive for over 2 years with serial testing at ~4 month intervals remained asymptomatic for the duration (20).

Asymptomatic infection has also been observed in shingleback lizards (*T. rugosa*) infected with SBNV. In animals presenting to a wildlife care facility, SBNV was detected by qRT-PCR in 12% of apparently healthy individuals (4 of 33) (21). Similarly, virus was detected on oral/choanal swabs (5 of 6) from “healthy” adult and subadult VCSV PCR positive chameleons (*C. calyptratus*) that did not develop respiratory disease over a 3 month period of monitoring prior to euthanasia (23). In freshwater turtles (*M. georgesi*) an intensive field survey was undertaken 6 months after the cessation of the initial BRV outbreak and BRV was detected by qRT-PCR in 29% (9 of 31) of apparently healthy individuals (22). It is likely as a result of ongoing development of targeted diagnostics, increased accessibility and affordability of NGS and a “virus exploration” approach (14) that additional nidoviruses will be detected in reptile species without clinical disease. The ongoing challenge will be to determine the clinical significance of these viruses unless detections are associated with investigations of natural outbreaks of disease or experimental infection trials are undertaken.

## Co-Infections

A co-infection, defined as more than one pathogen infecting an individual, is not uncommon in reptiles (64). To date coinfections with bacteria (15, 17, 55, 59), parasites (21, 45), and other viruses with nidoviral infection including snake retroviruses (18, 41) and an orthoreovirus (23) have been reported. Reptiles are known to harbour a wide range of normal resident microflora that can vary with the reptile species and the anatomical area of interest (65, 66). Therefore, interpretation of culture results, especially of the upper respiratory tract, must consider both the sampling methods, the clinical condition of the individual and evidence of associated pathology. Gramme-negative bacteria are commonly cultured from reptiles with acute or chronic respiratory disease but also from healthy animals (67). However, bacterial colonisation of the lower respiratory tract would be generally be considered a finding of significance (68). There is some evidence that secondary bacterial infections can contribute to the severity and clinical progression of reptile nidovirus infections (20). However, the interaction between nidoviral infection and other opportunistic pathogens, and the potential impact on morbidity and mortality, is an area for further investigation.

Genetically divergent reptile nidoviruses have also been identified in a single animal. Two reptile nidovirus sequences that shared only 71% global nucleotide identity have been reported in a python (20). A similar finding has also been reported in a veiled chameleon (*C. calyptratus*). These viruses only shared 53% nucleotide identity of ORF 1b, which is considered the most conserved region of the genome (23). Both the python and chameleon infected with more than one nidovirus died while exhibiting signs of respiratory disease. Nevertheless, in general, the clinical significance of infection with more than one nidovirus in reptiles remains largely uncertain.

## Transmission

The natural route(s) of transmission of nidoviruses in reptiles remains unclear. The successful infection of several *P. regius* with BPNV was achieved following oral and upper respiratory tract exposure (55). Subsequently, virus was detected in oral secretions and faeces of exposed animals. Multiple transmission routes are possible, including faecal-oral, fomite, and aerosolization. This is supported by detection of virus in respiratory epithelium, tissues of the gastrointestinal tract and on various antemortem swabs (18, 22). In reptiles generally, additional transmission trials are needed to provide further insights into the possible mechanisms of horizontal transmission.

Vertical transmission has been reported with nidoviruses in other animal species including porcine reproductive and respiratory syndrome (PRRS), equine arteritis virus (EAV), and gill-associated virus (GAV) (69–71). In a limited capacity, vertical transmission has been investigated by testing the eggs (*n* = 26) and hatchlings (*n* = 18) of nidovirus positive python mating pairs. Eggs were “cleaned” by exposure to UV irradiation or a quaternary ammonium disinfectant and artificially incubated. Despite virus being detected in/on most eggs following hatching only a single offspring became infected. This infection was detected when the offspring was 8–12 months of age and had been sampled at 4-monthly intervals. The viral sequence was more similar to the infected male (>98%), than the female (84%) of the breeding pair raising the question of whether there had been true vertical transmission (20). To date, vertical transmission has not been explored under “natural” conditions.

## Laboratory Diagnosis

The diagnostic options currently available for the detection of reptile nidovirus infections largely reflect the methodology used for their discovery. To date, most methods of detection are directed at the detection of virus or its components with next generation sequencing (NGS) laying the foundation for the development of both real time and conventional reverse transcription polymerase chain reaction (RT-PCR) assays. Such molecular assays are the predominant diagnostic tool available. Other diagnostic tools include transmission electron microscopy (TEM), virus isolation in cell culture, immunohistochemistry (IHC), and *in situ* hybridisation (ISH). There are currently no assays available for the detection of nidovirus specific antibodies. Given the recent discovery of nidoviruses in reptiles there is limited validation, or standardisation of diagnostic methods, offering opportunities for future research.

### Next Generation Sequencing

NGS and viral metagenomics have been used to detect novel reptile nidoviruses from a range of samples including fresh tissues (lung, trachea, oral mucosa, oesophagus, spleen, liver, kidney, gastrointestinal tract, whole snake associated nematodes) and swabs in viral transport media (oral swabs). With advances in technology, partly as a result of the difficulties when undertaking virus isolation, most sequencing occurs on nucleic acid extracted directly from a tissue sample or swab (13, 15–17), with few instances where the sequencing was undertaken on tissue culture fluid following successful virus isolation (18, 22). NGS results have been infrequently confirmed with additional sequencing as a method of validating sequence assembly (16, 22).

### Polymerase Chain Reaction

Both conventional and quantitative reverse-transcription (qRT-PCR) assays have been used to detect reptile nidoviruses. To detect virus, PCR offers several advantages when compared to TEM, virus isolation in cell culture and ISH. This includes fast results and a high analytical sensitivity and, usually, specificity. One of the advantages of NGS and molecular assays such as PCR is that new assays can be designed and first evaluated *in silico* to optimise performance before being applied to routine testing. In addition to qualitative (positive/negative) results, quantitative PCR assays also provide insights into the amount of virus present which can be used to develop associations between the virus detected and a possible role as the cause of a disease process (16, 18, 22). For juvenile pythons experimentally infected with BPNV, viral loads monitored by qRT-PCR increased for the duration of the trial with a more significant increase observed at 4 weeks post exposure (55), confirming active virus replication.

Most PCR assays target the most conserved region of the virus, ORF 1a or ORF 1b, or the more variable region encoding for the spike protein (16, 19, 20, 22, 41, 55, 59). As we improve our understanding of reptile nidoviral diversity, the design of PCR assays may move toward being more broadly reactive to ensure the spectrum of different reptile nidoviruses is initially detected. Subsequently, these should be followed by the use of more specific assays to detect individual viruses. Conversely, such broadly reactive assays can sometimes have reduced sensitivity when compared to virus specific assays. Therefore, assay selection will be influenced by the question at hand, and screening with both broad and specific assays may be necessary.

Several ante-mortem sample types including swabs, tracheal washes, faeces, and blood samples have been successfully used for PCR (41, 55). For snakes, swabs include choanal, oral/oesophageal, and cloacal (55). For lizards, oral swabs have been used from shinglebacks and chameleons (21, 23). For turtles conjunctival, oral, and cloacal swabs have been used (22). Little is known regarding the occurrence, onset, and duration of viraemia in reptiles infected with nidoviruses. Initial largescale antemortem surveys in snakes suggest that detecting virus in blood samples is not as sensitive as detecting virus on antemortem swabs. Consequently, blood should not be used as a preferred PCR sample type (19). A number of fresh tissues collected at post-mortem have been used to detect nidoviruses including the trachea, oesophagus, lung, liver, kidney, heart, spleen, stomach, small and large intestine, bladder, brain, eye, and ovary (17, 22, 55, 56).

The long duration of infection and viral shedding that is apparent in reptiles infected with nidoviruses facilitates the application of PCR based virus detection and surveillance. A longitudinal survey of pythons in a single collection over 28 months revealed that infection with a nidovirus can be chronic and definitive evidence of viral clearance was not observed (20). However, negative results should be considered carefully as they may reflect poor swabbing technique, sample handling, a period of low or interrupted viral shedding, the limit of detection of the assay or clearance of the virus. To control for the negative impacts of suboptimal swabbing technique and reduced efficiency of RNA extraction, an internal control to detect host DNA could be considered. However, there can be challenges associated with the selection of a host DNA target when testing a diversity of animal species. Alternative and perhaps preferred options that control for sample processing and PCR workflow issues include spiking of extraction solutions with an irrelevant/exogenous internal positive control RNA (72, 73). In practical terms, generating confidence in an individual animal's status can be effectively improved with serial sampling, which can be readily achieved by using qRT-PCR.

### Transmission Electron Microscopy

This technology has been responsible for the detection of many viruses either in suspension or in sections of tissue. Although it has been in use for many decades, TEM finds merit in situations where currently available assays do not allow visualisation of virus morphology, tissue tropism, intracellular events associated with virus replication, assembly and release from cells. Consequently, TEM is a valuable tool for pathogenesis studies (74, 75). Transmission electron microscopy has been used to identify the unique morphology of nidoviruses within pulmonary epithelial cells (16) and in cell culture supernatants (18, 22). Visualisation of the size and shape of virion particles, including the presence of a lipid envelope decorated with spikes, can also guide the subsequent selection of diagnostic assays, but the technology does lack sensitivity. Many opportunities remain to visualise and describe other reptile nidoviruses.

### Virus Isolation

Although considered a “gold standard” for the laboratory diagnosis of viral diseases, when compared to options available for the testing of mammalian species, the choice of established continuous reptile cell lines is limited. Furthermore, consideration must be given to the cultural requirements, especially temperature, for both cell lines and virus replication due to the poikilothermic nature of reptiles (76). However, for reptile nidoviruses, a range of both continuous cell lines and primary cell cultures have been successfully used to isolate viruses.

Stenglein et al. (16) attempted unsuccessfully to isolate BPNV from frozen infected tissues in several snake cell lines including the boa constrictor kidney (boa constrictor JK) and viper heart (viper VSW and VH-2) cell lines (16) but Hoon-Hanks et al. (55) were subsequently able to isolate BPNV in a primary diamond python (*Morelia spilota spilota)* cell culture. Cultures were inoculated with viral transport media from an oral swab collected from a *P. regius* and maintained at 30°C (55). Dervas et al. (18) successfully isolated MVNV using primary cultures of green tree python (*M. viridis*) liver and brain cells. Cultures were inoculated with homogenates of lung tissue from diseased snakes and maintained at 30°C (18). This isolate was then inoculated onto selected brain, kidney and lung cell cultures of a *Boa constrictor* to assess susceptibility and obtain an isolate free of a retrovirus contaminant. Convincing virus replication was identified in the kidney and lung cell cultures. The infected cells were also stained using anti-MVNV nucleoprotein (N protein) anti-serum at 3 days post inoculation, with all cell types except brain cells shown to be permissive for MVNV (18). Blahak et al. (41) attempted unsuccessfully to isolate virus from green tree pythons (*M. viridis*) using suspensions of liver, lung, kidney and intestine inoculated onto viper heart cells (VH-2) at 29°C (41).

Isolation of reptile nidoviruses from lizards was attempted unsuccessfully using *Boa constrictor* kidney and diamond python (*Morelia spilota spilota)* heart cell cultures, and two cell lines: iguana heart (IgH2) and viper heart (VH-2). Cultures were inoculated with fresh-frozen tissue homogenate (oral mucosa, lung, trachea) from a single chameleon (C. *calyptratus*) and maintained at 30°C (23). In freshwater turtles BRV was successfully isolated from pooled homogenates of spleen and lung tissue from freshwater turtles. Despite attempts using hamster lung (HmLu-1), avian (CEF), fish (SB, FHM, SSN-1), reptile (VH-2), and mosquito (C6/36) cell lines, BRV was successfully isolated, perhaps unexpectedly, using monkey kidney cells (CV-1, BGM, and Vero) maintained at 25°C (22).

The successful use of both primary and continuous cell cultures to isolate reptile nidoviruses highlights the opportunities to explore the susceptibility various cell cultures to infection with reptile nidoviruses. The benefits of producing an isolate are numerous, including easier identification and characterisation of a virus, differentiation between viable and non-viable virus, and production of high concentrations of material to facilitate nucleic acid sequencing and to underpin transmission studies. Unfortunately, generating primary cell cultures is going to depend on the capacity and interest of individual researchers to develop primary cell cultures for their species of interest or explore the suitability of a wide range of established cell lines.

### Immunohistochemistry and *in situ* Hybridisation

IHC and ISH offer unique opportunities to demonstrate viral proteins or RNA within the observed pathology or tissues of interest. IHC has been successfully used to visualise reptile nidoviral proteins in affected tissues. Dervas et al. (18) produced a polyclonal rabbit antibody by immunising a rabbit with a purified recombinant nucleoprotein of MVNV to demonstrate the nidovirus N protein in tissue sections of lung and trachea (18).

ISH has also been used to confirm the presence of nidoviral RNA in lung lesions (15, 18). Bodowes et al. (15) targeted the RNA-directed RNA polymerase (RdRp) gene to detect virus in viable and degenerate respiratory epithelial cells of the trachea and pharynx but not any other tissues from the python (15). Dervas et al. (18) performed ISH on the lungs of all affected snakes plus all major organs or tissues from five affected animals. In respiratory tissues virus was detected in the cytoplasm of pneumocytes lining the faveolar space and degenerated tracheal and nasopharyngeal epithelium. Viral RNA was also found within a few macrophages in the focal granulomatous-necrotising nephritis of a single snake but was not detected in other tissues including the stomach or intestines of infected snakes (18). In freshwater turtles, ISH was used to detect the gene encoding for the membrane protein (M) of BRV on a selection of tissues including in areas of necrotizing inflammation within the lacrimal gland, residual glandular epithelial cells, degenerate, or necrotic renal tubule epithelial cells and a foci of vasculitis. Viral RNA was also found in a dense focus of necrotizing cystitis, in scattered granulocytes in the urothelium and occasional granulocytes in the myocardial interstitium (22). When investigating disease outbreaks or undertaking pathogenesis studies the direct detection of viral RNA or antigens adds weight toward establishing the role of a new virus in the pathology observed (77). However, these methods depend on the availability or production of specific reagents, particularly antisera and labelled probes. In the absence of a virus isolate, to develop an IHC capability, nucleic acid sequence data is needed firstly for cloning to produce antigens using recombinant DNA technology and then the immunisation of animals to produce either polyclonal or monoclonal antibodies. ISH also depends entirely on the availability of nucleic sequence data but the development and synthesis of probes is much more prescriptive than the development of reagents for IHC.

### Serology

Serology often provides the missing link in disease investigations or establishing epidemiological patterns of disease. However, suitable tests are dependent on the nature and quality of the host immune response. Despite the diversity in reptiles, the study of the reptile immune system has generated one common conclusion: each taxa's immune response consists of a strong, broad, innate response, followed by a moderate specific immune response (78–80). Serological assays rely on the capacity of the host to develop and produce a detectable antibody response which may vary with a specific pathogen and reptile species (81). It can also be affected by other factors including temperature, reproductive status, seasonality, and stress or cortisone levels (78, 80). Serological assays have been developed for various tobaniviruses affecting cattle, pigs and horses (82, 83). However, there are no published serological assays for reptile nidoviruses. Serological assays have been developed for several other pathogens in reptiles with mixed success. Commonly used assay designs include virus neutralisation tests (VNTs) (84, 85), haemagglutination inhibition assays (HI) (86, 87), and enzyme-linked immunosorbent assays (ELISAs) (88, 89) although other designs have also been used.

A common difficulty in the development ELISA and similar assays is the limited availability of either broadly reactive or species-specific anti-reptile immunoglobulins, leaving researchers to develop reagents for their species of interest (90, 91) or design assays to avoid the requirement of such reagents. HI tests and VNTs do not suffer from these limitations but usually require whole virus and for VNTs appropriate cell cultures as well as an isolate. Current laboratory diagnosis of reptile nidoviruses relies heavily on PCR based detection, which is useful in detecting acute, chronic, or persistently infected animals, yet detecting antibodies as a means of indicating previous exposure remains a significant knowledge gap. The development of a serological assay can also complement a transmission trial through the detection of a humoral immune response to the virus. Intermittent viral shedding and varying viral loads in naturally infected animals (20) also highlights the need for a serological assay, despite the likely challenges, to provide alternative diagnostic approaches, especially for live animals.

## Management of Reptile Nidovirus Infection

Mortality rates in nidovirus infected reptiles can be significant. In a single collection of captive pythons, 75% (30 of 40) of infected animals died over a 28 month period (20) and there is strong indirect evidence BRV was associated with a significant population decline in an endangered turtle population (22). This decline is estimated to be more than 90% using population estimates generated years prior to the mortality event (47, 92, 93), however the population size of *M. georgesi* immediately prior to the outbreak is unknown. Specific antiviral treatments for nidovirus infected reptiles have not yet been reported and evidence to support other therapeutic treatments is limited. Reptiles are known to harbour a large range of normal microflora (64, 65, 94), and their role as a primary or secondary pathogen as part of a multifactorial respiratory syndrome can provide a target for supportive treatments (95). Antimicrobials, antifungals, antiprotozoals, anti-inflammatories, immunomodulators (e.g., parapox ovis virus immunomodulator) and supportive care (hydration, nebulization) have been used, however definitive peer-reviewed studies on the efficacy of various treatments has not been undertaken to date (16, 20, 21, 41, 56). Adequate light and heat are also fundamental aspects of supportive care known to impact on the overall health and immune response of reptiles (81). Furthermore, the factors that contribute to disease and the long-term survival following recovery from acute infection offers an opportunity for further investigation in both captive and wild reptile populations.

Management practises that limit the introduction and transmission of a reptile nidovirus in captive collections are consistent with general recommendations for hygiene and biosecurity in all facilities that house reptiles. Quarantining new animals from collection for a designated period to enable appropriate health cheques and screening of pathogens is strongly recommended (96). The duration of this period is often debated and is influenced by the knowledge of pathogens for that species (96). In light of the apparent long duration of reptile nidovirus infection in snakes (20) and as research continues into the field of reptile nidoviruses infecting lizards and turtles recommendations for different species may need to be revised. To date, effective strategies that have successfully prevented infection rates rising in a captive snake collection include quarantine of new or infected individuals and a separate caretaker, clothes, equipment, and separate ventilation for infected snakes. Additional measures included shower-out procedures, one-way flow of food and bedding, changing of disposable gloves between groups or species, hand sanitizer disinfection of gloves between breed rotations in racks, and disinfection of all surfaces and instruments following use (20). Management of reptile nidovirus infections in wild reptile populations has not yet been explored. In the absence of such specific data or proven recommendations, appropriate general biosecurity practises at national and international levels should be implemented (97, 98).

## Discussion

A review of nidoviruses in reptiles reveals a relatively new field of research. The literature is dominated by the detection of novel nucleic acid sequences that broaden our understanding of viral diversity in reptiles. Opportunities exist to further our understanding of this diversity, especially in the lesser studied species by opportunistic screening of samples from disease investigations, or PCR based surveys of wild reptile species. There also remains exciting knowledge gaps to fill, especially in the linking of novel sequences to clinical disease and pathology through transmission trials and fulfilment of Koch's postulates in various species. This will also explore the complexities of the likely routes of transmission. As more sequences are detected further research into determining if there are clear host-specific lineages, and the apparent resistance of different reptile species to infection will provide additional insights into host susceptibility. Opportunities to explore the impact of reptile nidovirus genotypes and co-infections on pathogenesis or virulence are also numerous.

Improvements in sequencing technology and analysis underpin the rapid development of targeted PCR assays. However, given the diversity of viruses detected in different species there may be significant advantages in moving toward the initial use of assays that are more broadly reactive to ensure different reptile nidoviruses or strains with minor genetic variation are not missed. The apparent long duration of nidoviral infection and shedding in snake species facilitates the use of PCR based monitoring. Conversely, asymptomatic infections, intermittent shedding, and varying viral loads, highlight the need for a serological assay to provide alternative diagnostic and surveillance approaches, especially for live animals. However, the development of such assays may prove challenging.

The study of nidoviruses in reptile populations has been focused on research in captive populations. The nature of captive reptile collections (the acquisition of wild and exchange of captive reptiles) can provide an opportunity for the introduction of both known and unknown pathogens. High holding densities and high rates of transfer between collections can potentially increase pathogen exposure and lower barriers to transmission (16). Furthermore, the escape or intentional release from captivity, especially of invasive species, could provide the opportunity for a pathogen to enter a naïve wild reptile population and cause significant mortality and morbidity. The illegal international trade of rare, unique and/or range restricted species also risks the introduction of both known or unknown pathogens into naïve reptile populations (99).

Unfortunately, the prevalence and distribution of nidoviruses in wild reptile populations is still largely unknown. In the midst of a global SARS-CoV-2 pandemic, the importance of understanding wildlife disease not only for the species involved but also for the potential public health implications, is readily apparent. By their nature, detecting emerging or novel pathogens is difficult, yet with advancing detection methods it is imperative that research into the extent and distribution of reptile nidoviruses continues so that we preserve the priceless biodiversity and critical reptile populations worldwide.

## Author Contributions

KP and LS conceived and designed this review. KP wrote the manuscript, analysed the data, and prepared figures and tables. EA, PK, and LS contributed to the concept and reviewed drafts of the manuscript. All authors contributed to the article and approved the submitted version.

## Funding

This review was supported by the College of Public Health, Medical, and Veterinary Sciences Higher Degree Research Enhancement Scheme, James Cook University, Australia.

## Conflict of Interest

The authors declare that the research was conducted in the absence of any commercial or financial relationships that could be construed as a potential conflict of interest. The handling editor declared a past co-authorship with one of the authors, EA.

## Publisher's Note

All claims expressed in this article are solely those of the authors and do not necessarily represent those of their affiliated organizations, or those of the publisher, the editors and the reviewers. Any product that may be evaluated in this article, or claim that may be made by its manufacturer, is not guaranteed or endorsed by the publisher.
