# Supplementary material for: Nidoviruses in Reptiles: A Review
Source: Front Vet Sci. 2021 Sep 21;8:733404. doi: 10.3389/fvets.2021.733404 (PMC8490724; doi:10.3389/fvets.2021.733404)
Supplement: Supplementary file 1 [file Table_1.pdf]

Supplementary Material

Supplementary Table 1: Nidovirus Sequences in reptiles identified in GenBank (>10,000 bp) by host used to generate a phylogenetic tree in Figure 2. The following search terms were used to identify these sequences; “*Tobaniviridae*”, “*Serpentovirinae*”, “unclassified *Nidovirales*”, “unclassified *Torovirinae*” and “unclassified *Serpentovirinae*”.

Host	Virus Name	GENBANK accession	Genome Coverage	Size (nt)	Reference
**Pythonidae**					
Ball Python (*P. regius*)	*Ball python nidovirus*	KJ541759	Complete genome	33, 452	(Stenglein et al., 2014)
	Ball python nidovirus 1 isolate 148	MG752895	Complete genome	33, 576	(Hoon-Hanks et al., 2018)
	Serpentovirinae sp. isolate L1	MN161567	Complete coding genome	31, 148	(Hoon-Hanks et al., 2019)
	Carpet python nidovirus 1 strain F17-605-16	MK722371	Complete genome	32, 319	(Dervas et al., 2020)
	Carpet python nidovirus 1 strain F17-700-16	MK722374	Complete genome	33, 200	(Dervas et al., 2020)
	Carpet python nidovirus 1 strain F17-750-56	MK722375	Complete genome	33, 531	(Dervas et al., 2020)
Green Tree Python *(M. viridis)*	*Morelia viridis nidovirus*	MF351889	Complete genome	32, 399	(Dervas et al., 2017)
	Morelia viridis nidovirus isolate BH171/14-7	MK182569	Complete genome	32, 753	(Blahak et al., 2020)
	Morelia viridis nidovirus isolate BH128/14-12	MK182566	Complete genome	32, 887	(Blahak et al., 2020)
	Morelia viridis nidovirus 2 strain F17-700-11	MK722372	Complete genome	31, 998	(Dervas et al., 2020)
	Serpentovirinae sp. isolate A93	MN161558	Complete coding genome	23, 843	(Hoon-Hanks et al., 2019)
	Serpentovirinae sp. isolate A94	MN161559	Complete coding genome	27, 626	(Hoon-Hanks et al., 2019)
	Serpentovirinae sp. isolate A95	MN161560	Complete coding genome	32, 375	(Hoon-Hanks et al., 2019)
	Serpentovirinae sp. isolate F17	MN161563	Complete coding genome	30, 797	(Hoon-Hanks et al., 2019)
	Serpentovirinae sp. isolate L4	MN161569	Complete coding genome	32, 953	(Hoon-Hanks et al., 2019)
	Serpentovirinae sp. isolate L8	MN161570	Complete coding genome	31, 911	(Hoon-Hanks et al., 2019)
	Serpentovirinae sp. isolate L3	MN161568	Complete coding genome	33, 115	(Hoon-Hanks et al., 2019)
	Carpet python nidovirus 1 strain F17-605-7	MK722366	Complete genome	34, 320	(Dervas et al., 2020)

	Carpet python nidovirus 1 strain F18-152-21	MK722378	Complete genome	31,149	(Dervas et al., 2020)
	Carpet python nidovirus 1 strain F16-37	MK722365	Complete genome	32,393	(Dervas et al., 2020)
	Carpet python nidovirus 1 strain F17-700-14	MK722373	Complete genome	32,498	(Dervas et al., 2020)
	Carpet python nidovirus 1 strain F18-15-liv	MK722376	Complete genome	33, 275	(Dervas et al., 2020)
	Carpet python nidovirus 1 strain F18-15-snt	MK722377	Complete genome	33, 550	(Dervas et al., 2020)
	Carpet python nidovirus 1 strain F17-605-8	MK722367	Complete genome	33, 604	(Dervas et al., 2020)
	Carpet python nidovirus 1 strain F16-11	MK722364	Complete genome	33, 604	(Dervas et al., 2020)
	Carpet python nidovirus 1 strain F17-605-10	MK722369	Complete genome	33, 620	(Dervas et al., 2020)
	Carpet python nidovirus 1 strain S15-1547	MK722379	Complete genome	34, 320	(Dervas et al., 2020)
Indian Python (*P.molorus*)	*Indian python nidovirus/ Python nidovirus isolate S1536/13*	KJ935003	Partial genome	33, 571	(Bodewes et al., 2014)
Blood Python (*P. brongersmai*)	*Serpentovirinae sp. isolate H0-1*	MN161564	Complete coding genome	33,381	(Hoon-Hanks et al., 2019)
	Serpentovirinae sp. isolate H0-2	MN161565	Complete coding genome	32,405	(Hoon-Hanks et al., 2019)
Woma Python (*A. ramsayi*)	*Serpentovirinae sp. isolate L14*	MN161571	Complete coding genome	24,392	(Hoon-Hanks et al., 2019)
	Carpet python nidovirus 1 strain F16-0171	MK722380	Complete genome	33,063	(Dervas et al., 2020)
Reticulated Python (*M. reticulatus*)	*Serpentovirinae sp. isolate K48*	MN161566	Complete coding genome	27,281	(Hoon-Hanks et al., 2019)
Carpet Python (*M. spilota*)	*Carpet python nidovirus 1 strain F17-0003*	MK722363	Complete genome	31,920	(Dervas et al., 2020)
	Carpet python nidovirus 1 strain F17-605-9	MK722368	Complete genome	33, 620	(Dervas et al., 2020)
Angolan Python (*P. anchietae*)	*Carpet python nidovirus 1 strain F17-605-12*	MK722370	Complete genome	33, 732	(Dervas et al., 2020)
**Boidae**					
Emerald tree boa (*C. caninus*)	*Serpentovirinae sp. isolate C18*	MN161561	Complete coding genome	27, 270	(Hoon-Hanks et al., 2019)
	Serpentovirinae sp. isolate C19	MN161562	Partial coding genome	11, 111	(Hoon-Hanks et al., 2019)
**Colubridae**					
Pope's keelback (*H. popei*)	*Hainan hebius popei torovirus LPSC33749*	MG600028	Complete coding genome	29, 409	(Shi et al., 2018)
Red Banded Snake (*L. rufozonatus*)	*Guangdong red-banded snake-Lycodon rufozonatus-torovirus LPSF30546*	MG600030	Complete coding genome	30, 859	(Shi et al., 2018)
Honduran milk snake	*Serpentovirinae sp. isolate L25*	MN161572	Complete coding genome	30, 104	(Hoon-Hanks et al., 2019)

(*L. t. hondurensis*)					
Mandarin Rat Snake (*E. mandarinus*)	*Guangdong mandarin rat snake torovirus*	MG600031	Partial genome	26, 031	(Shi et al., 2018)
**Homalopsidae**					
Chinese water snake (*M. chinensis*)	*Guangdong Chinese water snake torovirus*	MG600029	Partial genome	16, 038	(Shi et al., 2018)
**Nematoda sp found in snakes**					
Snake-associated nematodes mix Xinzhou [Nematoda sp. (14), Ascarididae sp.(2)]	*Xinzhou nematode virus 6*	KX883637	Partial genome	25, 960	(Shi et al., 2016)
Snake-associated nematodes mix Xinzhou [Nematoda sp. (14), Ascarididae sp.(2)]	*Xinzhou toro-like virus*	KX883638	Complete coding genome	30, 353	(Shi et al., 2016)
**Chamaeleonidae**					
Veiled chameleon (*C. calyptratus*)	*Veiled chameleon serpentovirus A*	MT997159	Complete coding genome	31, 537	(Hoon-Hanks et al., 2020)
	Veiled chameleon serpentovirus B	MT997160	Complete coding genome	36, 144	(Hoon-Hanks et al., 2020)
**Scincidae**					
Wild Shingleback Lizard (T. rugosa)	*Shingleback nidovirus 1*	KX184715	Partial genome	23, 832	(O'Dea et al., 2016)
**Chelidae**					
Bellinger River Snapping Turtle (*M. georgesi*)	*Bellinger River snapping turtle virus J248*	MF685025	Complete genome	30, 742	(Zhang et al., 2018)

References

Blahak, S., Jenckel, M., Höper, D., Beer, M., Hoffmann, B., and Schlottau, K. (2020). Investigations into the presence of nidoviruses in pythons. *Virology Journal* 17(1)**,** 6. doi: 10.1186/s12985-020-1279-5.

Bodewes, R., Lempp, C., Schürch, A.C., Habierski, A., Hahn, K., Lamers, M., et al. (2014). Novel divergent nidovirus in a python with pneumonia. *Journal of General Virology* 95(Pt 11)**,** 2480-2485. doi: 10.1099/vir.0.068700-0.

Dervas, E., Hepojoki, J., Laimbacher, A., Romero-Palomo, F., Jelinek, C., Keller, S., et al. (2017). Nidovirus-associated proliferative pneumonia in the green tree python (Morelia viridis). *Journal of Virology* 91(21). doi: 10.1128/JVI.00718-17.

Dervas, E., Hepojoki, J., Smura, T., Prähauser, B., Windbichler, K., Blumich, S., et al. (2020). Python nidoviruses, more than respiratory pathogens. *bioRxiv*.

Hoon-Hanks, L.L., Layton, M.L., Ossiboff, R.J., Parker, J.S.L., Dubovi, E.J., and Stenglein, M.D. (2018). Respiratory disease in ball pythons (Python regius) experimentally infected with ball python nidovirus. *Virology* 517**,** 77-87. doi: 10.1016/j.virol.2017.12.008.

Hoon-Hanks, L.L., Ossiboff, R.J., Bartolini, P., Fogelson, S.B., Perry, S.M., Stöhr, A.C., et al. (2019). Longitudinal and Cross-Sectional Sampling of Serpentovirus (Nidovirus) Infection in Captive Snakes Reveals High Prevalence, Persistent Infection, and Increased Mortality in Pythons and Divergent Serpentovirus Infection in Boas and Colubrids. *Frontiers in Veterinary Science* 6**,** 338. doi: 10.3389/fvets.2019.00338.

Hoon-Hanks, L.L., Stöhr, A.C., Anderson, A.J., Evans, D.E., Nevarez, J.G., Díaz, R.E., et al. (2020). Serpentovirus (Nidovirus) and Orthoreovirus Coinfection in Captive Veiled Chameleons (Chamaeleo calyptratus) with Respiratory Disease. *Viruses* 12(11)**,** 1329.

O'Dea, M.A., Jackson, B., Jackson, C., Xavier, P., and Warren, K. (2016). Discovery and partial genomic characterisation of a novel nidovirus associated with respiratory disease in wild shingleback lizards (Tiliqua rugosa). *PLoS ONE* 11(11)**,** e0165209. doi: 10.1371/journal.pone.0165209.

Shi, M., Lin, X.-D., Chen, X., Tian, J.-H., Chen, L.-J., Li, K., et al. (2018). The evolutionary history of vertebrate RNA viruses. *Nature (London)* 556(7700)**,** 197-202. doi: 10.1038/s41586-018-0012-7.

Shi, M., Lin, X.-D., Tian, J.-H., Chen, L.-J., Chen, X., Li, C.-X., et al. (2016). Redefining the invertebrate RNA virosphere. *Nature* 540(7634)**,** 539-543. doi: 10.1038/nature20167.

Stenglein, M.D., Jacobson, E.R., Wozniak, E.J., Wellehan, J.F.X., Kincaid, A., Gordon, M., et al. (2014). Ball python nidovirus: A candidate etiologic agent for severe respiratory disease in Python regius. *mBio* 5(5)**,** e01484. doi: 10.1128/mBio.01484-14.

Zhang, J., Finlaison, D.S., Frost, M.J., Gestier, S., Gu, X., Hall, J., et al. (2018). Identification of a novel nidovirus as a potential cause of large scale mortalities in the endangered Bellinger River snapping turtle (Myuchelys georgesi). *PLoS ONE* 13(10)**,** e0205209. doi: 10.1371/journal.pone.0205209.
